# Induced Human Decidual NK-Like Cells Improve Utero-Placental Perfusion in Mice

**DOI:** 10.1371/journal.pone.0164353

**Published:** 2016-10-13

**Authors:** Ricardo C. Cavalli, Ana Sofia Cerdeira, Elizabeth Pernicone, Henri A. Korkes, Suzanne D. Burke, Augustine Rajakumar, Ravi I. Thadhani, Drucilla J. Roberts, Manoj Bhasin, S. Ananth Karumanchi, Hernan D. Kopcow

**Affiliations:** 1 Department of Medicine, Beth Israel Deaconess Medical Center, Harvard Medical School, Boston, MA, United States of America; 2 Gulbenkian Programme for Advanced Medical Education, Lisbon, Portugal; 3 Howard Hughes Medical Institute, Chevy Chase, MD, United States of America; 4 Division of Nephrology, Massachusetts General Hospital, Harvard Medical School, Boston, MA, United States of America; 5 Department of Pathology, Massachusetts General Hospital, Harvard Medical School, Boston, MA, United States of America; Queen's University, CANADA

## Abstract

Decidual NK (dNK) cells, a distinct type of NK cell, are thought to regulate uterine spiral artery remodeling, a process that allows for increased blood delivery to the fetal-placental unit. Impairment of uterine spiral artery remodeling is associated with decreased placental perfusion, increased uterine artery resistance, and obstetric complications such as preeclampsia and intrauterine growth restriction. *Ex vivo* manipulation of human peripheral blood NK (pNK) cells by a combination of hypoxia, TGFß-1 and 5-aza-2’-deoxycytidine yields cells with phenotypic and *in vitro* functional similarities to dNK cells, called idNK cells. Here, gene expression profiling shows that CD56^Bright^ idNK cells derived *ex vivo* from human pNK cells, and to a lesser extent CD56^Dim^ idNK cells, are enriched in the gene expression signature that distinguishes dNK cells from pNK cells. When injected into immunocompromised pregnant mice with elevated uterine artery resistance, idNK cells homed to the uterus and reduced the uterine artery resistance index, suggesting improved placental perfusion.

## Introduction

Abnormal placentation sets the stage for the development of pregnancy complications that may present with devastating maternal and fetal outcomes. The mechanisms that regulate placental development remain elusive. Natural killer (NK) cells at the maternal-fetal interface are increasingly recognized as important players in this process[1,2].

Human NK cells are lymphocytes characterized by high cytolytic potential against tumor-transformed and virus-infected cells. Peripheral blood NK cells (pNK) represent ~10% of all circulating lymphocytes and are constituted mainly by CD56^Dim^CD16^+^ (CD56^Dim^ pNK) and a minor proportion of CD56^Bright^CD16^-^ cells (CD56^Bright^ pNK)[3,4]. At the maternal-fetal interface, NK cells accumulate in the specialized endometrium, the uterine decidua, where they peak by the end of the first trimester of pregnancy representing 70% of local lymphocytes. Decidual NK cells (dNK) are a different subset with unique properties that distinguishes them from pNKs [5,6]. dNKs are CD56^Bright^CD16^-^, but display markers that are not present in pNKs such as CD9 and CD49a. Most importantly dNKs express Killer cell Immunoglobulin-like Receptors (KIRs) and, even though they are granular and have perforin and granzymes, they are poorly cytotoxic [7]. In addition, dNKs secrete proangiogenic and trophoblast migration promoting factors[6,8–11]. dNK cells are in direct contact with trophoblast cells and are thought to regulate their invasion[6,11,12] and remodeling of uterine spiral arteries [11,13,14]. During normal pregnancy, uterine spiral arteries undergo morphological changes that result in increased diameter, wall thinning, and lower resistance [15], ultimately allowing for a more than 10-fold progressive increase in uterine blood flow [16] necessary to accommodate the demands of a growing fetal-placental unit. At the cellular level, spiral artery remodeling is characterized by replacement of endothelial cells by invading trophoblasts alongside with fibrinoid deposition and loss of elastic lamina and vascular smooth muscle cells[17].

Defective spiral artery remodeling leads to decreased placental perfusion and has been associated with pregnancy complications such as preeclampsia, intrauterine growth restriction (IUGR), and miscarriages [18–20]. These pathological conditions lead to significant maternal and fetal morbidity and mortality [21,22] and to date, there are no effective treatments.

Doppler interrogation of uterine arteries is used in the evaluation of placentation as an indicator of uteroplacental vascular resistance and attendant perfusion [23]. Importantly, increased uterine artery resistance has been confirmed by Doppler studies in clinical conditions associated with defective placentation [18,24,25] and is being extensively assessed as a screening tool for prediction of preeclampsia, IUGR and other pregnancy complications [26–29].

Animal studies have helped elucidate the role of NK cell regulation in spiral artery remodeling. Alymphoid mice (no NK, T or B cells) generated by double knock out of lymphoid recombinase activating 2 (Rag2) and common γ chain (γc) genes, *Rag2*^*-/-*^*γc*^*-/-*^ mice, show thickening of the spiral arteries with luminal narrowing that is reverted upon reestablishment of the NK cell population [30]. The narrow spiral arteries of this model parallel what is observed in human preeclampsia. Importantly, this model has been previously shown to have altered change in the hemodynamic profile of uterine arteries [31]. Although *Rag2*^*-/-*^*γc*^*-/-*^ mice do not present with hypertension or growth restriction as noted in severe preeclampsia [21,32,33], they constitute a representative model to examine the early stages of preeclampsia development. Because these immunodeficient mice are good recipients for xenogeneic cells, they are also a good model to test the ability of human cells to remodel uterine spiral arteries.

Human genetic studies have supported an association between NK cell biology and disorders of deep placentation in humans. Certain combinations of maternal NK cell KIR haplotypes (KIR AA, characterized predominantly by inhibitory receptors) and paternal HLA-C alleles expressed on trophoblast cells (HLA-C2) are associated with an increased risk of developing preeclampsia, IUGR or miscarriage [34,35].

We have previously shown that a combination of 5-aza-2’-deoxycytidine, hypoxia and TGFß-1 converted human pNK cells into NK cells with similarities to dNK cells (termed idNK cells) [36]. Here we demonstrate that idNK cells are enriched in the gene expression signature that differentiates dNK and pNK cells, and that idNK cell injection into pregnant immunocompromised mice reduces uterine artery resistance.

## Materials and Methods

Human samples were collected according to protocols approved by the Institutional Review Board at the Beth Israel Deaconess Medical Center. No consent form was required as data were analyzed anonymously and decidual samples are considered discarded material.

### Isolation of Human pNK cells

Human pNK cells were enriched from leukopacks using a negative enrichment Ab cocktail (NK RosetteSep, StemCell Technologies, Vancouver, BC, Canada) following the manufacturer’s instructions. For microarray analysis CD3^-^ CD56^Bright^ CD16^-^ pNK and CD3^-^ CD56^Dim^ CD16^+^ pNK cells were further purified by FACS sorting. Alternatively enriched pNK cells were seeded at 1 X 10^6^ cells/ml in IL-15 complete media (RPMI 1640 media containing 10% FCS, 1 U/ml penicillin, 100 mg/ml streptomycin, 2 mM L-glutamine, 1 mM sodium pyruvate, nonessential amino acids, and 55 nM 2-ME [all from Life Technologies, Grand Island, NY] with the addition of 10 ng/ml recombinant human IL-15 [PeproTech, Rocky Hill, NJ]) and maintained in culture in humidified incubators at 37°C with 21% O_2_ and 5% CO_2_ for 7 days (for injection at gd9) or 10 days (for injection at gd12) to be used as controls in animal experiments.

### Conversion of pNK cells to idNK cells

Conversion of pNK cells to idNK cells were done as previously described [36]. Briefly, NK RosetteSep enriched pNK cells were seeded at 1 X 10^6^ cells/ml in IL-15 complete media with the addition of recombinant human TGF-β1 (PeproTech) at 2 ng/ml, 1 μM 5-aza-2-deoxycytidine (Aza) (Sigma-Aldrich, St Louis, MO), and maintained in culture 7 days (for injection at gd9) or 10 days (for injection at gd12) at 37°C in humidified incubators in a 1% O_2_ and 5% CO_2_ atmosphere. To assess conversion efficiency, the percentage of CD9^+^ KIR^+^ cells among the CD16^-^ CD56^Bright^ and CD16^+^ CD56^Dim^ idNK populations was evaluated by flow cytometry after 6 or 7 days in culture. For microarray gene expression profiling, CD3^-^ CD16^-^ CD56^Bright^ CD9^+^ KIR^+^ idNK and CD3^-^ CD16^+^ CD56^Dim^ CD9^+^ KIR^+^ idNK cells were further purified from the idNK cell bulk preparation by FACS.

### Isolation of Human dNK cells

Human dNK cells for gene expression profiling were prepared from decidual tissue of first trimester elective terminations as previously described [36]. Briefly decidual tissue was minced with scissors and digested with 0.1% collagenase type IV and 0.01% DNAse I (both from Sigma-Aldrich, St. Louis, MO) 30 minutes at 37°C. The digestion was stopped by the addition of RPMI 1640 media containing 10% Fetal Bovine Serum. The digested tissue was then passed sequentially through 100μm, 70μm and 40μm cell strainers (BD Falcon, San Diego, CA) and washed with complete RPMI 1640 media, to obtain a single cell suspension. Cells were plated and incubated up to 2 hrs at 37°C to remove adherent cells. Decidual lymphocyte were then prepared from the overlaying cell suspension by density gradient centrifugation (Ficoll-Hypaque; GE Healthcare Life Sciences, Pittsburgh, PA). Cells were stained and sorted by FACS to isolate dNK cells defined as CD3^-^ CD56^Bright^ CD16^-^ lymphocytes.

### Microarray gene expression profiling

Flow-sorted cells were washed with cold PBS and cell pellets frozen and stored at -80C. Total RNA was isolated using the RNeasy mini kit from Qiagen, according to manufacture’s instructions. RNA was analyzed for integrity in the Agilent BioAnalyzer—RNA Pico and samples with integrity number > 6.5 were used to generate microarray data (n = 3 per group). Poly-A was added to RNA using the Gene Chip WT Plus Reagent Kit from Affymetrix (Santa Clara, CA) according to manufacturer’s instruction. Fragmented and labeled ss-DNA was then hybridized into Human Transcriptome Array (HTA 2.0) from Affymetrix. HTA is a most comprehensive array that allows profiling of coding genes, long-noncoding RNA and transcript isoforms. Microarray analysis was conducted by the Genomics, Proteomics, Bioinformatics and Systems Biology Center at the Beth Israel Deaconess Medical Center according to the standard Affymetrix protocol using the high-throughput Affymetrix system. The quality of hybridized chips was assessed using Affymetrix guidelines on the basis of PM mean, 3' to 5' ratios for beta-actin and GAPDH and values for spike-in control transcripts. Reproducibility of the samples was checked by using chip to chip correlation and signal-to-noise ratio (SNR) methods for replicate arrays using arrayQualityMetrics, a Bioconductor package in R [37]. All the high quality arrays were included for unsupervised and supervised bioinformatics analysis. Expression data for 70,523 core transcripts were preprocessed using the Robust Multichip Average (RMA) method in R using the Bioconductor and associated packages. RMA performed background adjustment, quantile normalization and final summarization of oligonucleotides per transcript using the median polish algorithm [38]. Probes located on Y-chromosome as well as with undetectable expression intensity were removed from further analysis. Raw microarray data was deposited in NCBI GEO Database (GSE85592)

#### Unsupervised analysis

The unsupervised analysis was performed using Principal Component Analysis (PCA), which projects multivariate data objects onto a lower dimensional space while retaining as much of the original variance as possible [39,40].

Unsupervised analysis was also performed using hierarchical clustering analysis (HCA). For HCA, Pearson correlation test with ward-linkage method was used to cluster samples from various biological groups.

#### Identification of differentially expressed genes

To identify differentially expressed genes, a linear model was implemented using linear model microarray analysis software package (limma)[41]. *Limma* estimates the differences between experimental and control groups by fitting a linear model and using an empirical Bayes method to moderate standard errors of the estimated log-fold changes for expression values from each probe set. The differentially expressed probes were identified on the basis of absolute fold change and Benjamini and Hochberg corrected P value [42].

#### Gene Set Enrichment analysis (GSEA)

GSEA was performed using the GSEA-R, a Bioconductor implementation of GSEA from Broad Institute [43]. The GSEA was performed to compare pNK CD56^Bright^ vs. idNK_CD56^Bright^ transcriptome signatures with pNK_CD56^Bright^ vs. dNK transcriptome signatures, or pNK_CD56^Dim^ vs. idNK CD56^Dim^ transcriptome signatures with pNK CD56^Dim^vs dNK transcriptome signatures. These analyses assist in statistical determination as to whether idNK CD56^Bright^ and idNK CD56^Dim^ have acquired a transcriptome signature similar to dNK cells from pNK CD56^Bright^ and pNK CD56^Dim^ respectively. Before GSEA, normalization expression data and generation of transcriptome signature were performed using the approach described under “Microarray gene expression profiling” section. Analysis was run with 1000 geneset based permutations to perform enrichment analysis. The significance enrichment was determined on the basis of normalized enrichment score (NES) and Nominal P value (P value).

#### Pathway Enrichment analysis

The Ingenuity Pathway Analysis (IPA 8.0) (Qiagen) was used to identify the pathways significantly affected by genes that are altered in various comparisons. The knowledge base of this software consists of functions, pathways and network models derived by systematically exploring the peer-reviewed scientific literature. A detailed description of IPA analysis is available at the Ingenuity Systems’ web site (http://www.ingenuity.com). It calculates p-value for each pathway according to the fit of user’s data to the IPA database using one tailed Fisher exact test. The pathways with multiple test corrected p-values <0.001 were considered significantly affected.

### Multiplex ELISA

Supernatants of idNK and control pNK cells, the later cultured in parallel under 21% O_2_ in IL-15 complete media, from three donors, were harvested from 7-day cultures and frozen at -80C. pNK secretion baseline was established by culture of pNK cells from three independent donors for 24 hrs in media containing IL-15 at 21%O_2_. Frozen supernatants were thawed and evaluated by Luminex kit MILLIPLEX® MAP Human Cytokine/Chemokine Panel 1 (EMD Millipore) for cytokines RANTES, EGF, Eotaxin-1, G-CSF, GM-CSF, IFN-2α, IFN-γ, IL-1α, IL-1ß, IL-1R1, IL-2, IL-3, IL-4, IL-5, IL-6, IL-7, IL-8, IL-10, IL-12 p40, IL-12, IL-13, IL-15, IL-17A, IP-10, MCP-1, MIP-1α, MIP-1ß, TNF-α, TNF-ß, VEGF following manufacturer instructions. Supernatants were frozen again at -80C and evaluated 5 days later with MILLIPLEX® MAP Human Angiogenesis/Growth Factor Configurable 17 Plex Panel (EMD Millipore) for analytes Angiopoietin-2, BMP-9, EGF, Endoglin, Endothelin-1, FGF1, FGF2, Follistatin, G-CSF, HB-EGF, HGF, Leptin, PLGF, VEGF, VEGF-C, VEGF-D. Measurements were done with a Luminex XMAP Technology MAGPIX System version 4.2 (Life Technologies) using xPONENT software. Values were exported to Microsoft Excel for analysis and statistics. Values from control media were subtracted from each measurement and groups compared by paired t-test.

### Animal Experiments

All animal studies were approved by the Beth Israel Deaconess Medical Center Institutional Animal Care and Use Committee. Balb/c (BALB/cAnNCrl) mice were purchased from Charles River Laboratories International, Inc. (Wilmington, MA, United States). A local Balb/c Rag2^*-/-*^γc^*-/-*^ (BALB/c-Rag2^*-/-*^γc ^*-/-*^) colony was established with animals kindly provided by Drs Anne Croy and Chandrakant Tayade (Queen’s University, Kingston, ON, Canada).

Ten weeks old Balb/c and Balb/c Rag2^*-/-*^ γc ^*-/-*^ [44,45] females were mated with males of their same strain. The day a vaginal plug was detected was considered gestational day (gd) 0. Pregnant Balb/c mice and a group of pregnant Balb/c Rag2^*-/-*^ γc ^*-/-*^ females were monitored by Doppler ultrasound at gd12 and gd17 to calculate uterine artery resistance index. Three additional groups of Balb/c Rag2^*-/-*^ γc ^*-/-*^ received vehicle (PBS), 15 x 10^6^ idNK cells or 15 x 10^6^ pNK cells maintained in culture with 10 ng/mL IL-15 for 7 days, in 150μl of PBS by tail vein injection on gd9. For injection, mice were anesthetized with 3.5% isoflurane in oxygen administered via a precision vaporizer. At gd12 mice were subjected to Doppler ultrasound examination to measure uterine artery resistance index, followed by administration of a second dose of cells from the same donor or vehicle alone. At gd17 mice were re-evaluated by Doppler US and euthanized under 4% isoflurane, and blood samples were collected by left ventricular puncture. Hearts and uteri were collected. Hearts were weighed. Individual implantation sites were dissected. Average fetal weight was calculated measuring the weight of all fetuses from each litter together and dividing the weight by the number of fetuses. Average placental weight was calculated dividing the weight of 3 or 4 placentas per female and dividing the weight by the number of placentas weighted. Embryo resorption rates were calculated by dividing the number of resorpted implantation sites by the total number of implantation sites per animal and averaging the results for each group. After removal of fetuses, some implantation sites were fixed in 4% paraformaldehyde for 12 hours and then placed in 70% ethanol for paraffin imbedding for histology.

#### Uterine artery resistance index determination by Doppler Ultrasound

Mice were initially anesthetized in a closed chamber with oxygen and approximately 3% isoflurane and placed on a heated stage to maintain core temperature at 37°C. Anesthesia was maintained with approximately 1.5% isoflurane via a nose cone. Fur was removed from the abdominal region. Heart rate, respiratory frequency and temperature were monitored throughout the procedure. Pre-warmed gel for sonography was used as a coupling medium. A 48 MHz transducer operating at 100 fps was used to transcutaneously image spiral arteries. The transducer was fixed in the abdominal region and after identification of the bladder was angled at 30° to identify the right uterine artery behind the bladder arising from the common iliac by color Doppler. Doppler mode, pulse repetition frequency was set between 4 and 48kHz to detect low to high blood flow velocities, respectively. A 2 to 4mm pulsed Doppler gate was used and the angle of the Doppler beam and the vessel was recorded and kept <60°. After obtaining five similar waveforms with good image quality the image was frozen. Peak systolic velocity (PSV) and end diastolic velocity (EDV) were measured. The same procedure was performed for the left uterine artery and measured two times for each side. The procedure was performed by a team of 2 individuals. When one was operating the transducer, the other performed the necessary steps to freeze images and make measurements. The RI of the right and left uterine arteries were calculated by the formula RI = (PSV-EDV)/PSV and averaged. The ultrasound parameters obtained included fetal heart rate, temperature and uterine artery pulse wave Doppler. Measurements were done with a Vevo® 2100 (VisualSonics) platform.

#### Tracking of injected idNK cells

Bulk idNK cell preparations were labeled with 1 μM of fluorescence dye “cell-tracker red” CMTPX (Molecular Probes^®^ –Life Technologies, Grand Island, NY, USA) for 15 min at 37°C followed by 30 minutes incubation in pre-warmed complete RPMI media (without IL-15) following manufacturer’s instructions. The cells were injected at doses of 2 x10^6^, 5 x10^6^ and 15 x10^6^ cells by tail vein injection in pregnant Rag2^*-/-*^ γc ^*-/-*^ females at gd9 and mice euthanized at gd12. Non-pregnant mice received 15 x10^6^ cells and were euthanized three days post injection. Liver, lung, spleen and implantation sites were collected, embedded in O.C.T Compound Medium (Tissue-Tek® 4583) and immediately placed at -80°C. Frozen blocks were stored at -20C. 7 mm sections were cut using cryostat (Shandon Cryotome, Thermo Scientific®) at -20°C and placed on adhesion slides (Superfrost Plus, Thermo Scientific®). Images were acquired with a fluorescence microscope (Olympus BX41—Cellsens® Standard—Life Science Imaging Software). Images from the DAPI, FITC, and Texas Red channels were obtained and overlaid.

#### Histology of uterine spiral arteries

Paraformaldehyde and ethanol fixed implantation sites were cut in half using a razor blade and embedded in paraffin. 7 μm thick sections were mounted on glass slides and stained with hematoxylin and eosin. Two sections per implantation site 42 μm apart were examined. All transversally cut spiral arteries located in the decidua were identified in each section and marked on low magnification pictures of each section by a placenta pathologist blinded to samples identity. Lumen perimeter and muscular wall perimeter of those arteries were delineated and measured under 20x objective magnification by a double blinded observer using Olympus BX41 and Cellsens® Standard—Life Science Imaging Software. Lumen diameter (LD) was calculated from the lumen circumference (LC) as follows (LD = LC/π), wall diameter (WD) was calculated as follows (WD = WC/π) where WC is the muscular wall circumference, considering π = 3.141592. Wall to lumen ratio was calculated as WD / LD, Wall thickness was calculated as (WD-LD) / 2. Two implantation sites per mouse for each group (idNK cell injected, IL-15 pNK cell injected, each n = 6 pregnant females per group, and vehicle control injected, n = 7 pregnant females) were evaluated. Histological sections of two of these implantation sites presented insufficient transversally cut decidual vessels and were therefore excluded from the analysis. In one implantation site, only one section was measured as the other section was <42 μm apart. The number of spiral arteries sections measured were 90 for the vehicle control group, 85 for the IL-15 treated NK cell control group, and 60 for the idNK treated group.

### Flow Cytometry Studies

For analytical Flow Cytometry or preparative FACS sorting the following fluorescently labeled antibodies were used: anti-CD56 (clone B159), anti-CD16 (clone 3G8), anti-CD3 (clone SK7), anti-CD9 (clone ML-13), anti-CD49a (clone SR84), anti-CD151 (clone 14A2.H1), anti–granzyme A (clone CB9), anti-perforin (clone dG9), anti–granzyme B (clone GB11),and isotype controls were all from BD Biosciences (San Diego, CA). Cell surface molecule staining and intracytoplasmic staining were done as previously described[36]. KIR expression was evaluated with a cocktail of four PE-conjugated antibodies: anti-CD158b, anti-CD158a, anti-NKB1 and anti-NKAT2 all from BD Biosciences. Preparative FACS sorting was done using a FACS Aria cell sorter (Becton Dickinson). Analytical flow cytometry was performed on a BD FACSCanto flow cytometer (Becton Dickinson). Chemokine receptor staining was done as previously described[36] using the following fluorescently labeled mAbs or corresponding isotype controls: anti-CXCR4 (clone 12G5), anti-CXCR1 (clone 42705), anti-CXCR3 (clone 49801), anti-CCR7 (clone 150503) (all from R&D Systems), and anti-CX3CR1 (clone 2A9-1; MBL International, Woburn, MA). Data from CD56Dim idNK cells not previously shown is compared here to that of CD56Bright idNKs.

## Results

### Gene expression profiling of idNK cells

**Fig 1:**
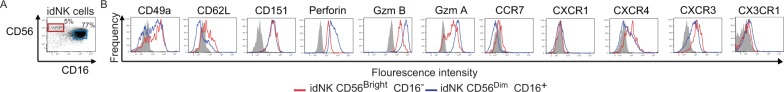
CD56^Dim^ and CD56^Bright^ idNK cells express a panel of markers that differentiate dNK cells from pNK cells with a similar expression pattern. (A) Representative flow cytometry dot plot of idNK cell preparations showing CD56^Bright^ CD16^-^ (red gate) and CD56^Dim^ CD16^+^ (blue gate) populations. Plots are gated on CD3^-^ cells. (B) Representative histograms showing expression of markers by CD56^Bright^ CD16^-^ (red) and CD56^Dim^ CD16^+^ (blue) idNK cells. Gray histograms correspond to isotype control staining of CD56^Bright^ CD16^-^ idNK.

Exposure of pNK cells to a combination TGF-ß1, hypoxia and 5-aza-2’-deoxycytidine (Aza) yields idNK cells that similar to dNK cells express CD9 and KIRs, secrete proangiogenic molecules and have reduced cytotoxicity[36]. CD56^Bright^ idNK cells express a panel of molecules and chemokine receptors in a pattern similar to dNK cells and distinct from CD56^Bright^ pNK as evaluated by flow cytometry. These included CD49a, CD62L, CD151, Perforin, Granzyme A and Granzyme B, CCR7, CXCR1, CXCR4, CXCR3 and CX3CR1[36]. Interestingly, a similar expression pattern for these molecules was also acquired by CD56^Dim^ idNK cells (**Fig 1**).

**Fig 2:**
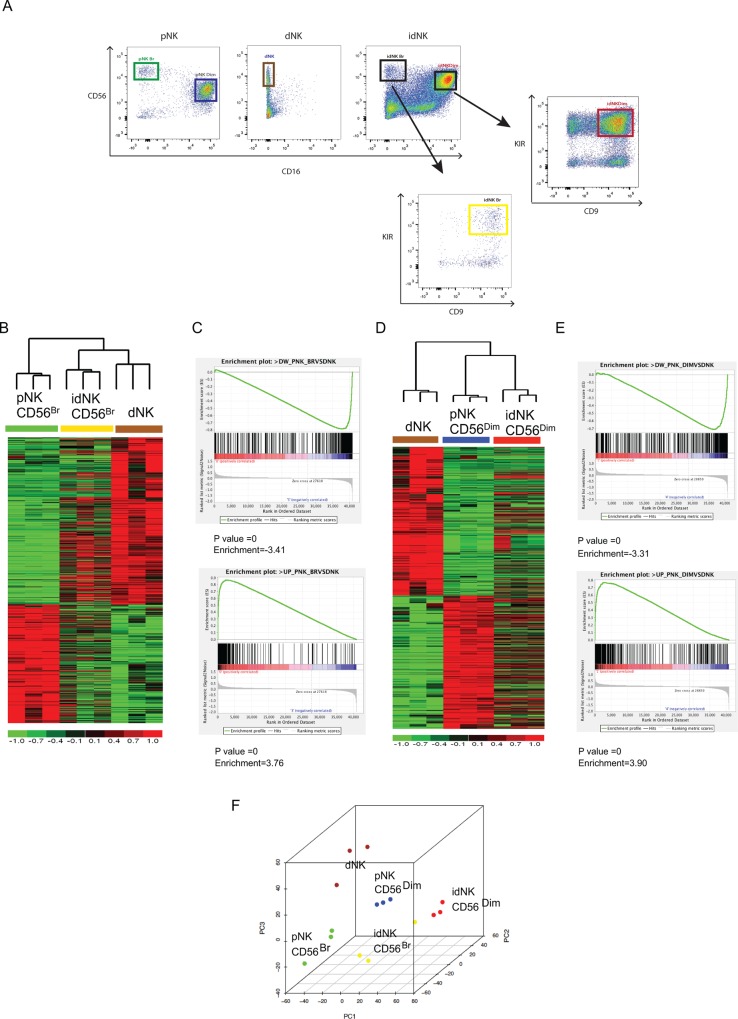
Characterization of idNK cells by transcriptome profiling. (A) Gating strategy used to FACS sort dNK, pNK CD56^Bright^, pNK CD56^Dim^, idNK CD56^Bright^ and idNK CD56^Dim^ (both idNK populations sorted as CD9^+^ KIR^+^ cells), used for microarray gene expression profiling. Plots are gated on CD3^-^ cells. Heatmap of genes differentially expressed (Fold change > 2 and FDR < 0.05) in pNK CD56^Bright^ vs. dNK (B) or in pNK CD56Dim vs. dNK (D). Heatmaps also include idNK CD56Bright (B) or idNK CD56^Dim^ samples (D) to explore correlation in their expression pattern with pNK CD56^Bright^ (B) or pNK CD56^Dim^ (D) and dNK samples. Columns represent samples, and rows represent genes, with expression in pseudocolor scale (–1 = green, +1 = red). C and E) Gene set enrichment analysis comparison of expression using geneset based permutations. Genes significantly downregulated in pNK CD56^Bright^ vs. dNK shown significant downregulation patterns in pNK CD56^Bright^ compared to idNK CD56^Bright^ (FDR P value = 0, Normalized enrichment score [NES] = 3.41) [C, Upper Panel]. Genes significantly upregulated in pNK CD56^Bright^ vs. dNK shown significant upregulation patterns in pNK CD56^Bright^ compared to idNK CD56^Bright^ (FDR P value = 0, NES = 3.76) [C, Lower Panel]. Genes significantly downregulated in pNK CD56 Dim vs. dNK did not show significant downregulation patterns in pNK CD56^Dim^ compared to idNK CD56^Dim^ (P value = 0, NES = 3.38) [E, Upper Panel]. Genes significantly upregulated in pNK CD56^Dim^ vs. dNK shown significant upregulation patterns in pNK CD56^Dim^ compared to idNK CD56^Dim^ (P value = 0, NES = 3.90) [E, Lower Panel]. (F) Unsupervised principal component analysis based on coding genes All analyses exclude Y chromosome encoded genes.

To further characterize the similarities between idNK and dNK cells, we compared the gene expression profile of FACS sorted CD16^-^ CD56^Bright^ idNK and CD16^+^ CD56^Dim^ idNK to that of dNK, CD16^-^ CD56^Bright^ pNK and CD16^+^ CD56^Dim^ pNK cells. Conversion of pNK cells to idNK cells is marked by the acquisition of CD9 and KIR expression[36], therefore only CD9^+^ KIR^+^ cells were collected from the CD56^Bright^ idNK and CD56^Dim^ idNK cell populations for gene expression profiling (**Fig 2A**).

Transcripts differentially expressed between the different cell types were identified from normalized and preprocessed data on the multiple test corrected P value, P value < 0.05, and absolute fold change > 2 among group pairs. Differentially expressed genes in each pair wise comparison are provided in **S1 File**. All analyses excluded Y chromosome encoded genes as all dNK samples were from female donors whereas the gender of pNK and idNK samples donors is unknown.

To evaluate how much of the gene expression signature that differentiates dNK cells from CD56^Bright^ pNK cells was acquired by CD56^Bright^ idNK cells, genes differentially expressed between dNK and CD56^Bright^ pNK were clustered based on the level of expression across dNK and CD56^Bright^ pNK samples. The heatmap was expanded by adding the expression level of corresponding genes in idNK CD56^Bright^ samples without reorganizing the gene clusters (**Fig 2B**). The gene expression pattern of idNK CD56^Bright^ samples closely resembled the dNK expression pattern. In agreement with this finding, hierarchical clustering based on these differentially expressed genes clustered idNK CD56^Bright^ samples in a branch adjacent to dNK samples and distant from pNK CD56^Bright^ samples (**Fig 2B**). 843 transcripts were differentially expressed with at least a 2 fold change and a False Discovery Rate (FDR) < 5% between dNK and CD56^Bright^ pNK cells (shown in blue in the graph of **S2 File**). Approximately 45% of these transcripts changed expression with at least 2 fold change and FDR<5% in CD56^Bright^ idNK cells from CD56^Bright^ pNK cells in the same direction (upregulated, or downregulated) as in dNK cells (**S2 File**). A detailed list of the 843 transcripts with the fold change in gene expression in CD56^Bright^ pNK vs. dNK (shown in blue bars) and CD56^Bright^ pNK vs. CD56^Bright^ idNK (shown in red bars if fold change was greater than 2 and FDR < 5%, or green bars if they did not meet these two criteria) is presented in **S2 File.** To further determine the significance of correlation in CD56^Bright^ pNK, idNK CD56^Bright^ and dNK transcriptome profiles, gene set enrichment analysis (GSEA) was performed [43]. For GSEA, genes significantly downregulated or upregulated in pNK CD56^Bright^ compared with dNK were used as geneset. The geneset contains 350 and 493 upregulated and downregulated genes respectively. The enrichment of this geneset was tested in the expression change in pNK CD56^Bright^ vs. idNK CD56^Bright^ and significance was tested on the basis of Normalized enrichment score and P value[43]. The GSEA depicted that the pNK CD56^Bright^ vs. dNK gene set was significantly correlated with expression changes (upregulation / downregulation) in pNK CD56^Bright^ vs. idNK CD56^Bright^ using geneset based (Downregulated genes NES = 3.41, P-Value = 0, Upregulated genes NES = 3.76, P-Value = 0, **Fig 2C** top and bottom panels respectively).

When similar analyses were carried out with CD56^Dim^ idNK, CD56^Dim^ pNK and dNK cell samples, based on genes differentially expressed between CD56^Dim^ pNK and dNK cells at least at a 2 fold change and FDR<0.05 (Gene list provided in **S2 File**), CD56^Dim^ idNK were more closely associated with CD56^Dim^ pNK cells than dNK cells by hierarchical clustering (**Fig 2D**). However GSEA using geneset based permutation[43] showed significant enrichment of the pNK CD56^dim^ vs. dNK upregulated gene set in pNK CD56^Dim^ vs. idNK CD56^Dim^ upregulated genes (NES = 3.90, P-Value = 0, **Fig 2E** lower panel) and genes downregulated in pNK CD56^Dim^ vs. dNK among genes downregulated in pNK CD56^Dim^ vs. idNK CD56^Dim^ (NES = 3.31, P-Value = 0, **Fig 2E** upper panel)

To establish the relationship between CD56^Bright^ idNK, CD56^Dim^ idNK, dNK, CD56^Bright^ pNK and CD56^Dim^ pNK cells populations unsupervised microarray data analysis was conducted considering the complete transcriptome. The principal component analysis (PCA) (**Fig 2F**) grouped samples according to the 5 population cell types, with one CD56^Bright^ idNK sample mapping close to the CD56^Dim^ idNK samples, and segregated samples along the primary component 1 (PC1) according to whether they were fresh *ex vivo* cells (dNK, CD56^Bright^ pNK and CD56^Dim^ pNK cells) or treated cells (CD56^Bright^ idNK and CD56^Dim^ idNK). In agreement with this observation, complete transcriptome unsupervised hierarchical clustering grouped idNK cell, both CD56^Dim^ and CD56^Bright^ in a separate branch from freshly isolated NK cells (**S1 Fig**).

Thus idNK cells are not true dNK cells as revealed by complete transcriptome unsupervised PCA and unsupervised hierarchical clustering. However CD56^Bright^ idNK are significantly enriched in the gene expression signature that differentiates dNK from CD56^Bright^ pNK, and CD56^Dim^ idNK are significantly enriched in gene signature that differentiates dNK cells from CD56^Dim^ pNK cells based on geneset based permutation GSEA results. However, in the case of CD56^Dim^ idNK, GSEA results are not consistent with the data obtained by signature-based hierarchical clustering that finds CD56^Dim^ idNK more closely associated with CD56^Dim^ pNK than with dNK cells.

#### Pathway enrichment analysis in idNK cells

**Fig 3:**
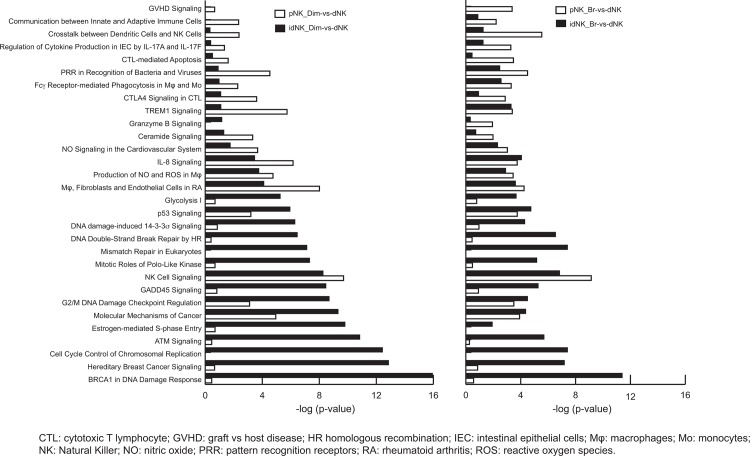
Canonical pathways enrichment analysis in idNK cells. Pathways enrichment analysis on significantly differentially expressed genes (Fold change>2 and FDR<0.05) identified in different comparisons (idNK_CD56^Dim^-vs-dNK, idNK_CD56^Bright^-vs-dNK, pNK CD56^Bright^-vs-dNK, pNK_CD56^Dim^-vs-dNK or pNK CD56Bright-vs-pNK CD56^Dim^) to determine the pathways acquired by idNK cells. 30 pathways differing in the level of significance in idNK CD56^Dim^ -vs-dNK and pNK CD56^Dim^-vs—dNK, or in idNK CD56^Bright^ -vs—dNK and pNKCD56^Bright^-vs-dNK are shown. Left panel: Level of significance of the affected pathways in pNK CD56^Dim^ (white) and idNK CD56^Dim^ (black) compared to dNK cells. Right panel: Level of significance of the affected pathways in pNK CD56^Bright^ (white) and idNK CD56^Bright^ (black) compared to dNK cells. Each bar represents a pathway with significance of enrichment determined using the Benjamini-Hochberg hypothesis corrected p-value. The P value is shown as–log P-value on the x-axis.

In order to identify dNK cell pathways or functions that were acquired by idNK cells (CD56^Bright^ or CD56^Dim^) and pathways that are different between dNK and idNK cells, pathway enrichment analysis was carried out using differentially expressed genes presenting at least a 2 fold change and FDR< 0.05. Overall analysis identified 136 pathways significantly affected (p-value < 0.001) in at least one of the following pair wise comparisons: idNK_CD56^Dim^ vs. dNK, idNK_CD56^Bright^ vs. dNK pNK_CD56^Bright^ vs. dNK, pNK_cD56^Dim^ vs. dNK or pNK_CD56^Bright^ vs. pNK_CD56^Dim^ (**S3 File**). Of these, 30 pathways differing in level of significance between idNK_CD56^Dim^ vs. dNK and pNK_CD56^Dim^ vs. dNK, or between idNK_CD56^Bright^ vs. dNK and pNK_CD56^Bright^ vs. dNK were selected (**Fig 3**). The P value is a significance level with smaller values (represented with longer bars in **Fig 3**) indicating increasing confidence in the effect on the pathway. In **Fig 3**white bars correspond to the comparison of pathways between fresh pNK cells (CD56^Dim^ left panel, or CD56^Bright^ right panel) to dNK cells, black bars represent the comparison of pathways between idNK cells (CD56^Dim,^ left panel or CD56^Bright^, right panel) and dNK cells. Most pathways that are highly affected in idNK cells compared to dNK cells but not in pNK vs. dNK cells are related to cell stress and proliferation (i.e. BRCA1 in DNA Damage Response, Cell Cycle Control of Chromosomal Replication, Cell Cycle: G2/M DNA Damage Checkpoint Regulation, Mismatch Repair in Eukaryotes, Glycolysis I, DNA damage-induced 14-3-3s Signaling, DNA Double-Strand Break Repair by HR) suggesting that culture conditions induced stress and idNK proliferation may explain some of the differences in gene expression between idNK and dNK cells. On the contrary, pathways related to immune function (i.e. GVHD Signaling, Communication between Innate and Adaptive Immune Cells, Crosstalk between Dendritic Cells and NK Cells, CTL-mediated Apoptosis, PRR in Recognition of Bacteria and Viruses, CTLA4 Signaling in CTL, Granzyme B Signaling) that were significantly different between dNK vs. pNK cells became more similar in idNK and dNK cells.

### idNK Cytokine secretion profiling

**Fig 4:**
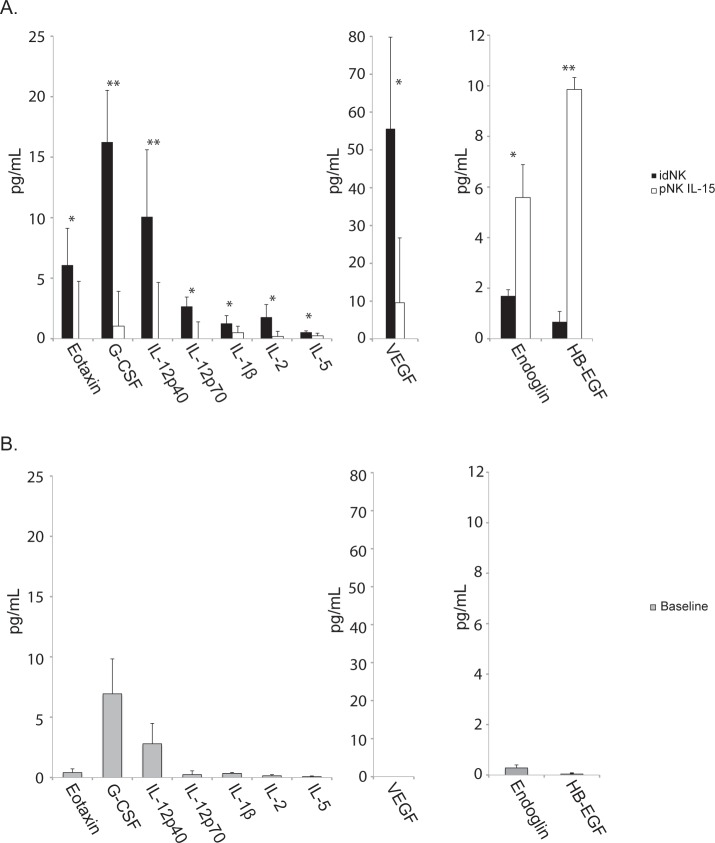
Cytokine, angiogenic factors and growth factors secretion profile of idNK cells. A) Supernatants from idNK and control pNK cell 7 days-long cultures were measured for a panel of 44 cytokines, angiogenic factors and growth factors from two overlapping luminex kits. Only analytes with statistical significant difference between the two groups are shown. Average of samples from three donors is shown. Error bars represent standard deviation. ** p < 0.01, *p < 0.05 by paired t-test. B) 24 hours pNK secretion baseline determined with supernatants from three independent donors. Error bars represent standard deviation.

dNK cells have been reported to secrete soluble factors relevant to uterine angiogenesis and spiral artery remodeling such as VEGF-A, VEGF-C, PLGF and IFNγ [6,8,46]. In order to identify cytokines secreted by idNK cells, 7-day long cell culture supernatants from idNK cells and control NK cells maintained in culture with IL-15 where analyzed by multiplex Elisa (**Fig 4A**). A 24 hours pNK secretion baseline was determined with supernatants from independent donors (**Fig 4B**). idNK cells secreted Eotaxin, G-CSF, IL-12p40, IL-12p70, IL-1ß, IL-2, IL-5 with statistical significance compared to control cells (**Fig 4**). As expected VEGF was secreted by idNK more than by control cells, however, its concentration varied with the type of multiplex kit used, in one case its concentration was 55pg/ml (**Fig 4**), while VEGF concentrations were 900 pg/ml when measured on the second multiplex platform. **(S1 Table).** Endoglin and HB-EGF levels were lower in idNK cells than in the control cells. A number of cytokines including EGF, GM-CSF, IFNγ. IL-13, IL-1Ra, IL-4, IP-10, MIP1-α, MIP1-ß, RANTES, TNFα, TNFß and PLGF were produced by both idNK cells and control NK cells when compared to pNK baseline levels, but without statistical significant differences between idNK and control groups. VEGF-C and VEGF-D were not detected in the supernatants (S1 Table).

### idNK cell injection improves uteroplacental hemodynamics in a mouse model with elevated uterine artery resistance

In addition to sharing the expression of many genes with dNK cells, idNK cells share functional properties with dNK cells in vitro [36]. A mouse model with narrow uterine spiral arteries was used to test if human idNK cells can correct uterine artery flow *in vivo*.

**Fig 5:**
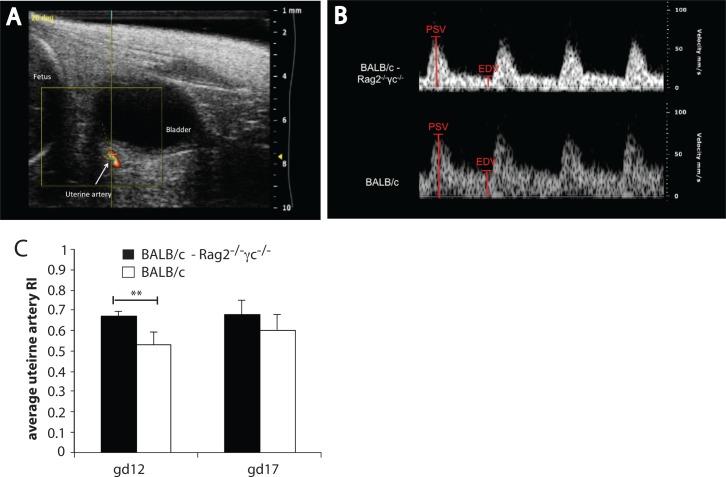
BALB/c Rag2^-/-^γc^-/-^ mice have higher uterine artery resistance than BALB/c mice. (A) Representative image of a uterine artery located behind the bladder by color Doppler Ultrasound. (B) Uterine artery flow velocity waveforms of BALB/c (bottom) and BALB/c Rag2^-/-^γc^-/-^(top) were obtained by pulse Doppler to measure the peak systolic velocity (PSV) and end diastolic velocity (EDV). (C) Average Uterine Artery resistance Index (RI) at gestational days 12 (gd12) and 17 (gd17) from 6 BALB/c and 5 BALB/c Rag2^-/-^γc^-/-^ mice. Error bars represent standard deviation. ** p-value = 0.0012 by two-tail non parametric t-Test.

Uterine spiral artery narrowing increases uteroplacental blood flow resistance in disorders of placentation, and is associated with elevated uterine artery resistance index (RI) detected by Doppler Ultrasound. Balb/c *Rag2*^*-/-*^ γc ^*-/-*^ pregnant mice showed elevated uterine artery RI compared to wild-type Balb/c pregnant controls by Doppler ultrasound at gd12 (p<0.01) and at gd17, although at gd17 differences were not statistically significant (**Fig 5**).

**Fig 6:**
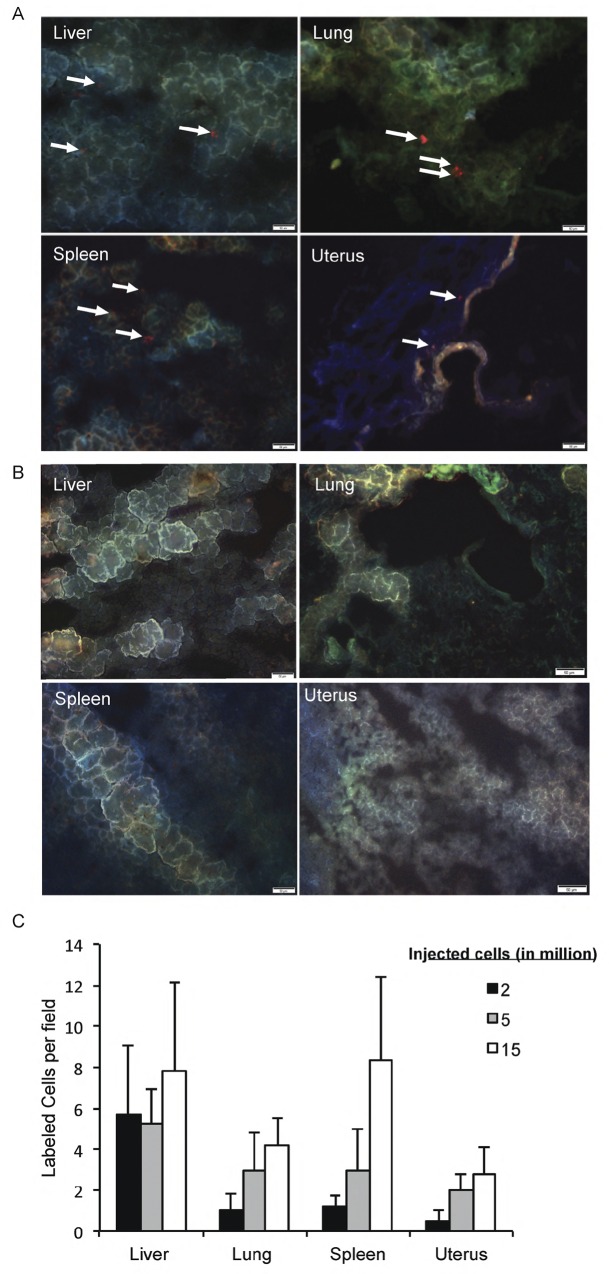
idNK cells can be found in the uterus of injected Rag2^-/-^ γc^-/-^ pregnant mice. A) Photomicrographs showing the presence of CellTrakerTM Red CMTPX labeled idNK cells in liver, lung, spleen and uterus of pregnant Rag2^-/-^γc^-/-^ females. Labeled idNK cells were injected through the tail vein on gd9 and organs harvested on gd12. White arrows point to labeled cells. (B) Representative photomicrographs of liver, lung, spleen, and uterus from non-injected control pregnant females at gd12. Bars represent 50μm. (C) Average number of labeled cells in 3 random fields per organ in frozen sections harvested gd12 from pregnant Rag2^-/-^ γc^-/-^ females injected with different doses of labeled idNK cells on gd9 are shown. The average of three random fields per organ is shown. Error bars represent standard deviation.

To test if idNK cells home to the uterus of pregnant *Rag2*^*-/-*^γc ^*-/-*^ mice, bulk idNK cells labeled with a red fluorescent dye were administered by tail vein injection into pregnant *Rag2*^*-/-*^ γc ^*-/-*^ females on gd9 and organs collected on gd12 to observe for the presence of red labeled cells by fluorescence microscopy in frozen sections. Red cells were observed in the liver, lung, spleen and implantation sites (uterus) of injected mice (**Fig 6A**) but not in control non-injected pregnant females (**Fig 6B**). A large number of labeled cells were retained in liver, lung and spleen. The number of cells homing to the uterus was lower and dose dependent, with 2 x 10^6^ or 5 x 10^6^ injected cells yielding few red cells in the uterus, while injection of 15 x 10^6^ cells resulted in visualization of a larger number of red cells in uterine sections (**Fig 6C**). Under the experimental conditions used idNK cells homed to the uterus of pregnant mice but not to the uterus of non-pregnant females. Red cells were observed in the liver but not in the uterus of non-pregnant injected mice (**S2 Fig)**. Some red spots observed in the uterus of non-pregnant females were the result of autofluorescence as the signal was also present on the FITC channel as well as in the uterus of non-injected non-pregnant animals (**S2 Fig)**.

**Fig 7:**
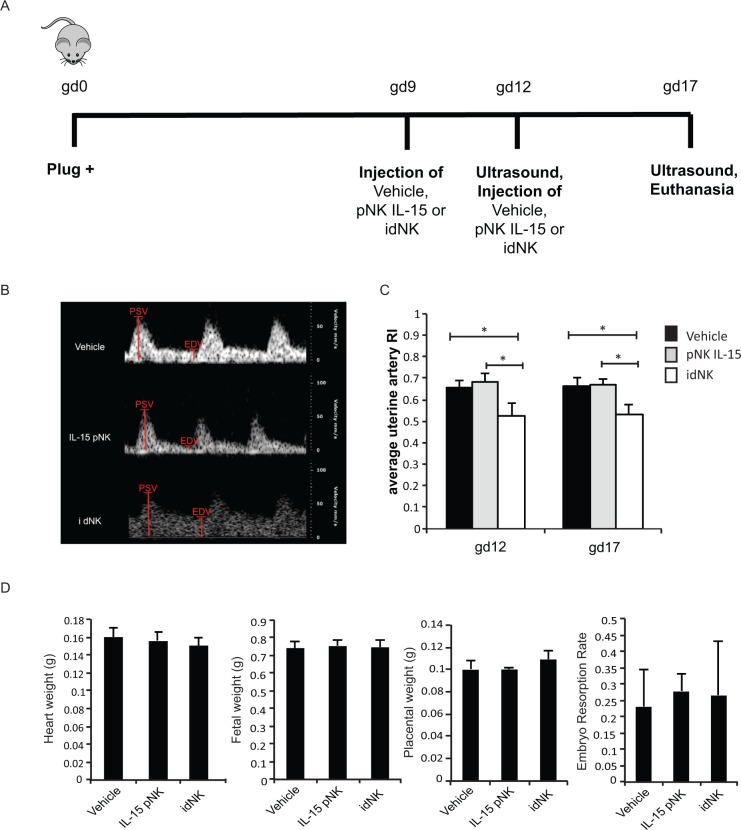
Injection of idNK cells improves uterine artery blood flow. (A) Outline of in vivo experimental design. Rag2^-/-^γc^-/-^ were mated and the day a vaginal plug was detected was considered gd0. At gd9 mice were injected with 15 million bulk idNK cells, pNK control cells maintained in culture with IL-15 under 21% O_2_, or vehicle alone (PBS) by tail vein injection. On gd12 left and right horn uterine arteries resistance indexes were evaluated by Doppler Ultrasound followed by the administration of a second dose of 15 million cells or vehicle. At gd17 ultrasound evaluation was repeated and mice sacrificed to collect implantation sites, heart and plasma. (B) Representative uterine artery flow velocity wave obtained by Doppler Ultrasound from uterine arteries at gd12 of animals injected with vehicle (top), control cells cultured in IL-15 (center) or idNK cells (bottom). Peak systolic velocity (PSV) and end diastolic velocity (EDV), shown in red, were measured and used to calculate uterine arteries resistance index (RI). (C) Average resistance index (RI) of the left horn and right horn uterine arteries at gd12 and gd17 of mice injected with vehicle, control pNK cells cultured in IL-15 or idNK cells. Average of 6 animals per group is shown. Error bars represent standard deviation. * p-value <0.05 by one way ANOVA with Bonferroni's Multiple Comparison Test. (D) Heart weight, Fetal weight, placental weight and embryo resorption rate at gd17. N = 6 animal per group, placental weight of idNK treated mice is the average of 7 animals. Comparisons were done by one way ANOVA with Bonferroni's Multiple Comparison Test.

**Fig 8:**
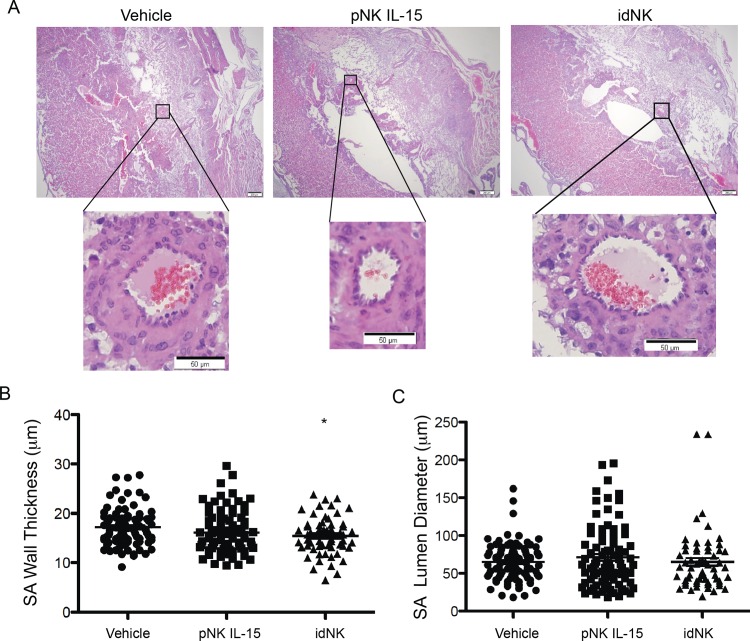
Effect of idNK injection on Rag2^-/-^ γc^-/-^ mice uterine spiral arteries. (A) Photomicrograph of representative implantation sites at gd17 of mice injected on gd9 and gd12 with vehicle (PBS), pNK control cells cultured in IL-15, or idNK cells (upper images, scale bar 200μm). Illustrative decidual spiral arteries transversal sections are shown in higher magnification (lower images, scale bar 50μm). Spiral arteries muscular wall thickness (B) and lumen diameter (C) from implantation sites of mice receiving the indicated treatments. Each circle, square or triangle corresponds to an individual spiral artery transversal section. Mean is indicated with a line. Measurements were obtained from one or two Hematoxylin Eosin stained implantation site sections separated 42 microns apart, from each of 2 implantation sites per pregnant female. idNK and pNK IL-15 treated groups consisted each of 6 females, vehicle control group consisted of 7 females. The number of spiral artery transversal sections evaluated for each group were: Vehicle, 90; pNK IL-15, 85, idNK, 60. * p<0.05 between idNK and Vehicle by one way ANOVA with Bonferroni's Multiple Comparison Test.

To evaluate if idNK cells can improve uterine artery hemodynamics, 15 x 10^6^ bulk idNK cells were injected intravenously in timed pregnant Rag2^*-/-*^ γc ^*-/-*^ females at gd9. At gd12 uterine artery resistance index was measured by Doppler ultrasound followed by the administration of a second dose of cells. At gd17 ultrasound measurements were repeated and mice euthanized to collect organs and implantation sites. Control groups received vehicle alone, or pNK cells maintained in culture with IL-15 (necessary to maintain NK cells alive) for the same time as idNK cells (**Fig 7A**). Efficiency of pNK to idNK cell conversion was evaluated before injection at gd9 measuring the percentage of CD9^+^ KIR^+^ cells among the CD56^Bright^ idNK and CD56^Dim^ idNK cell component of the idNK cell preparation by flow cytometry (**S2 Table**). Doppler ultrasound interrogation of uterine arteries showed that uterine artery resistance at gd12 and gd17 was significantly reduced (p<0.05) in animals receiving idNK cells but not in mice injected with control pNK cells maintained in culture with IL-15 (**Fig 7B and 7C**). Notably at gd12 the uterine artery RI was similar in idNK cell treated Rag2^*-/-*^ γc ^*-/-*^ mice and wild-type Balb/c mice (**Figs 7C and 5C**). Treatment with idNK or control cells did not produce statistically significant changes in heart weight, fetal weight or fetal resorption rates. Histological examination of implantation site deciduas revealed that spiral arteries wall thickness was not different in idNK cell treated animals compared to animals treated with control NK cells maintained in culture in IL-15. However, it was reduced with statistical significance when idNK cell treated mice were compared to vehicle treated controls. Differences between vehicle and control NK cell treated animals were not significant thus indicating a trend towards wall thickness reduction in idNK treated mice (**Fig 8A and 8B**). Spiral artery lumen diameter (**Fig 8C**) and wall to lumen ratio (not shown) were not significantly altered.

## Discussion

Previous phenotypical analysis and *in vitro* functional studies showed that idNK cells acquired specific markers expressed by dNK but not pNKs such as CD9 and CD49a, as well as the expression pattern of 14 other molecules [36]. Additionally, idNKs exhibited secretion of pro-angiogenic molecules and reduced cytotoxicity [36]. Here, we demonstrate by microarray gene expression profiling that idNKs are enriched in the gene expression signature that differentiates dNK cells from pNK cells. Importantly, when injected into a mouse model with defective spiral artery remodeling, idNK cells reduced uterine arteries RI suggesting they enhanced placental perfusion. In contrast, injection of control cells (cultured for 1 week in the absence of hypoxia, TGFß and AZA) did not induce any of these changes. Interestingly, the improvement in uterine artery RI was associated with a modest amelioration in spiral artery wall thickness in mice injected with idNK cells when compared to PBS injected controls.

Microarray gene expression profiling indicates that idNK cells are significantly enriched in the gene expression signature that differentiates dNK cells from pNK cells. Acquisition of the dNK signature seems to be more robust in CD56^Bright^ idNK cells from CD56^Bright^ pNK cells than in CD56^Dim^ idNK cells from CD56^Dim^ pNK cells. GSEA showed that CD56^Bright^ pNK vs. dNK gene set was significantly correlated with expression changes in CD56^Bright^ pNK vs. CD56^Bright^ idNK and hierarchical clustering based on genes differentially expressed between CD56^Bright^ pNK and dNK cells showed CD56^Bright^ idNK cells more closely associated to dNK cells, whereas changes in gene expression from CD56^Dim^ pNK to CD56^Dim^ idNK cells were less robust with GSEA showing significant enrichment of the dNK signature in CD56^Dim^ idNK samples but hierarchical clustering showed a closer association of CD56^Dim^ idNK samples with CD56^Dim^ pNK than with dNK samples.

Given the more robust dNK signature acquisition by CD56^Bright^ idNK injection of isolated CD56^Bright^ idNK cells should yield more effective results. However CD56^Bright^ idNK cells remain a minor population in idNK cultures and their numbers too low for injection as an isolated species. Attempts to establish the identity of uterine homing idNK cells as CD56^Bright^ or CD56^Dim^ by flow cytometry yielded no conclusive results.

Based on pathways enrichment analysis a number of immune function related pathways were more similar between dNK and both CD56^Dim^ and CD56^Brignts^ idNK cells, than between dNK and pNK cells. Crosstalk between NK cells and dendritic cells, and communication between innate and adaptive immune cells were among those pathways. It should be noted that although idNK cells are enriched in the dNK vs. pNK gene signature, idNK cells are not true dNK cells. Unsupervised principal component analysis based on the complete transcriptome segregated samples on the first component axis depending on whether they were fresh ex-vivo cells (dNK, CD56^Bright^ pNK and CD56^Dim^pNK) or *in vitro* treated cells (CD56^Bright^ idNK and CD56^Dim^ idNK). Consistent with these findings unsupervised hierarchical clustering grouped idNK cells in a branch separate from freshly isolated dNK and pNK cells. In accordance, pathways related to cell replication and stress were affected in idNK cells compared to dNK cells but not in pNK cells compared to dNK cells, indicating that cell proliferation and stress induced by culture conditions explain some of the differences existing between idNK and dNK cells.

As culture conditions may explain some differences in gene expression between idNK and freshly isolated NK cells, it is also possible that some of the gene expression differences between freshly isolated dNK and pNK cells presented here or previously described [5] arise from differences in the dNK and pNK isolation procedures that expose decidual cells but not pNK cells to tissue enzymatic digestion and in vitro culture at 37°C.

Administration of idNK cells into pregnant Rag2^*-/-*^γc ^*-/-*^ decreased uterine artery RI, combined with a trend towards decreased spiral artery wall thickness. The development of a low resistance uteroplacental circulation, associated with spiral artery remodeling is an important determinant of the uterine Doppler waveform in pregnancy, but is not the only one[47]. It is possible that the change in spiral arteries wall thickness might have influenced uterine artery RI, however, other factors such as the release of vasogenic mediators by idNKs, and other physiologic changes, should also be considered. For instance, uterine radial artery resistance index is correlated with the number of peripheral blood NK cells in patients with recurrent pregnancy loss [48]. Injection of idNK cells resulted in spiral arteries with reduced wall thickness compared to vehicle (PBS) control treated animals, however without statistical significance from control NK cells. One interpretation of these findings is that control NK cells have a modest effect on spiral artery remodeling and that this effect is more readily seen with idNK cells. Rag2^-/^γc ^-/-^ do not present altered pregnancy outcomes. Taken together with lack of changes in fetal weights or litter number, these data indicate that the effect of idNK cells in this mouse model is modest. It is possible that a larger effect may be observed in a model with a more severe phenotype.

In mice, uterine NK cells start to accumulate in the uterus around gd6, peak at gd10-12 and decline thereafter [49–52], spiral artery remodeling starts at day 9 and is completed by day 12.5[53,54]. Presumably injection of idNK cells at gd7 or earlier rather than at gd9, may be more efficient on uterine spiral arteries remodeling in this model. However the pNK to idNK conversion protocol involves culture of cells for 7 days, and therefore injection at earlier time points would require setting up cultures before knowing the mice are pregnant making the study impractical and costly. Improvements in the pNK to idNK conversion protocols may reduce cell culture times allowing for this kind of experiments.

Pregnant Rag2^*-/-*^ γc ^*-/-*^ mice do not reproduce the whole spectrum of preeclampsia, fetal growth restriction [21,32,33], infertility or miscarriage. It would therefore be important to test the functional significance of idNK cells in other animal models with more severe maternal or fetal adverse outcomes.

Further characterization and refinement of conversion may lead to an increased efficacy and reduction of the number of cells necessary for injection. Currently, the amount of blood necessary to isolate a sufficient number of NK cells is not practical for the translation of this model into the clinic. Additional molecules such as transcription factors, epigenetic regulators as well as a combination of other factors may increase the specificity and efficiency of conversion. Methodologies for cell expansion [55] are evolving at a fast pace and can reduce the number of cells that need to be isolated to achieve the desired results. Alongside, it is extremely important to restrict possible off-target effects. In the present study, fetal, placental and (maternal) heart weights and embryo resorption rate, were not different between idNK, control cell or vehicle injected mice. However, although a small number of injected idNK cells reached the uterus, most idNK cells were retained in the liver, lung and spleen, which may lead to undesired side effects. Invasive procedures during pregnancy, such as chorionic villous sampling and amniocentesis are currently performed in daily clinical practice. A modification of these could be developed to deliver idNKs directly into the decidua.

The robust reduction in uterine artery RI induced by idNK cells contrasts with the modest effect on SA remodeling and relatively small number of idNK cells recruited to the uterus. Perhaps earlier evaluation of idNK cell homing to the uterus. ie 24hs instead of 72hs post injection, would have yielded larger numbers. It is also possible that small changes in the many spiral arteries into which the uterine artery ramificates may translate into reduced RI in the stem uterine artery. Finally the effects on uterine artery RI may be caused by soluble factors released by idNK cells independently of their homing to the uterus or their effects on SA remodeling. Decidual NK cells have been reported to produce angiogenic factors such as VEGF-A, VEGF-C, IFNγ, or PLGF important for spiral artery remodeling and uterine angiogenesis. Our multiplex ELISA results showed that both idNK cells produce significantly more VEGF than control cells. However, other vascular factors such as IFNγ α nd PLGF were not differentially altered in idNK cells and other VEGF family members such as VEGF-C and VEGF-D were below the detection limit of the Luminex assays. It is worth noting that although in our hands highly purified dNK cells did not produce VEGF-A (it was necessary to culture them for longer times or under hypoxia for them to produce the molecule)[8,36] previous reports showing VEGF secretion by dNK cells[6] served to establish the rationale for a pNK to idNK conversion protocol, that yielded cells with biological effect on uterine artery resistance. Since VEGF secretion by dNK cells is variable based on in vitro conditions, future studies should evaluate the biological role for VEGF secretion by dNK cells in animal models where VEGF dosage could be precisely titrated.

idNK cells also produced G-CSF. G-CSF is produced by decidual NK cells [56] and has been used to try to improve implantation success in IVF patients lacking activating KIR receptors (2 DS 1, 2 DS 3 and 3 DS 5) with previous history of repetitive implantation failure or long-term unexplained sterility[57], and as a treatment for patients with recurrent miscarriages [58,59]. Furthermore, it has been proposed that G-CSF may be important throughout pregnancy [57–59]. Future studies should examine whether G-CSF or combined with other factors may be able to improve uterine artery flow and placentation.

idNKs cells partially revert their phenotype upon removal of idNK cell inducing conditions [36]. It may be desirable for idNKs to have a long lasting effect, independent of the microenvironment. Although idNK cells display a cytotoxicity level significantly lower than control cells, they still have cytotoxic capacity [36]. While in the mouse there were no obvious side effects, this deserves further attention.

In summary, defective placentation predisposes to preeclampsia, IUGR and other adverse outcomes. Conversion of human peripheral blood NK cells to idNK cells yielded cells with transcriptional, phenotypical and functional similarities to dNK cells, capable of improving uterine artery hemodynamics and presenting a trend towards improvement of uterine spiral arteries in a mouse model of defective spiral artery remodeling. Future studies in other models of placentation disorders with more severe phenotype are needed to expand on these findings to develop a potential NK cell-based approach for prevention and/or treatment of preeclampsia and related disorders of defective placentation.

## Supporting Information

S1 Fig Unsupervised hierarchical clustering of idNK, dNK and pNK samples Ward pearson unsupervised hierarchical clustering of dNK, CD56^Bright^ idNK, CD56^Dim^ idNK, CD56^Bright^ pNK, CD56^Dim^ pNK samples based on expression of coding genes excluding Y chromosome genes. (EPS)

S2 Fig idNK cells do not home to the uterus of non-pregnant Rag2^-/-^ γc^-/-^ mice. A) Photomicrographs of liver (A) and uterus (B) of non-pregnant Rag2^-/-^ γc^-/-^ mice injected (top) and non-injected (bottom) with 15 million CellTrakerTM Red CMTPX labeled idNK cells. Organs harvested 3 days post tail vein injection. Images obtained with a FITC filter (left), a Texas Red filter (center) and both channels superimposed (right). White arrows point to labeled cells. Bars represent 50μm.
(PDF)

S1 File Cell type pair-wise comparisons. List of genes differentially expressed with at least a two fold change and false discovery rate (FDR) of 0.05 in different pair-wise cell type comparisons.
(XLSX)

S2 File Comparison of gene expression changes between dNK and idNK cells from pNK cells. First tab—Transcripts differentially expressed with at least a 2 fold change and a FDR < 5% between dNK and CD56^Bright^ pNK cells (first tab). Graph shows change in gene expression in CD56^Bright^ pNK vs. dNK in blue bars, and CD56^Bright^ pNK vs. CD56^Bright^ idNK (shown in red bars if fold change was greater than 2 and FDR < 5%, or green bars if they did not meet these two criteria). Second Tab- Transcripts differentially expressed with at least a 2 fold change and a FDR < 5% between dNK and CD56^Dim^ pNK cells). Graph shows change in gene expression in CD56^dim^ pNK vs. dNK in blue bars, and CD56^Dim^ pNK vs. CD56^Dim^ idNK (shown in red bars if fold change was greater than 2 and FDR < 5%, or green bars if they did not meet these two criteria).
(XLSX)

S3 File Pathway enrichment analysis. 136 pathways significantly affected (P value < 0.001) in at least one of the following pair wise comparisons: idNK_CD56^Dim^ vs. dNK, idNK_CD56^Bright^ vs. dNK, pNK_CD56^Bright^ vs. dNK, pNK_CD56^Dim^ vs. dNK or pNK_CD56^Bright^ vs. pNK_CD56^Dim^. The P value is a significance level with smaller P values indicating increasing confidence in the effect on the pathway.
(XLSX)

S1 Table Complete cytokine secretion data of idNK cells. Supernatants from idNK and control pNK IL-15 collected at 7 days were measured for a panel of 46 cytokines, angiogenic factors and growth factors from two overlapping luminex kits. P-values shown are derived from two-tailed t-tests comparing the mean concentration of each analyte in supernatants of idNK cells vs. pNK cells from three donors. ** p < 0.01, *p < 0.05. pNK cells 24hs baseline expression correspond to fresh pNK cell from three independent donors cultured 24hs in the presence of IL15.
(PDF)

S2 Table pNK to idNK conversion efficiency of cell preparations injected in mice. idNK conversion efficiency was evaluated by the percentage of CD9^+^KIR^+^cells in CD3^-^CD56^Bright^CD16^-^ NK cell and in CD3^-^CD56^Dim^CD16^+^ cell populations in the culture. Mice were injected with cells from the donor indicated in the table.
(PDF)
