# Supplementary figures and images for: Induced Human Decidual NK-Like Cells Improve Utero-Placental Perfusion in Mice
Source: PLoS One. 2016 Oct 13;11(10):e0164353. doi: 10.1371/journal.pone.0164353 (PMC5063315; doi:10.1371/journal.pone.0164353)

A Injected Liver

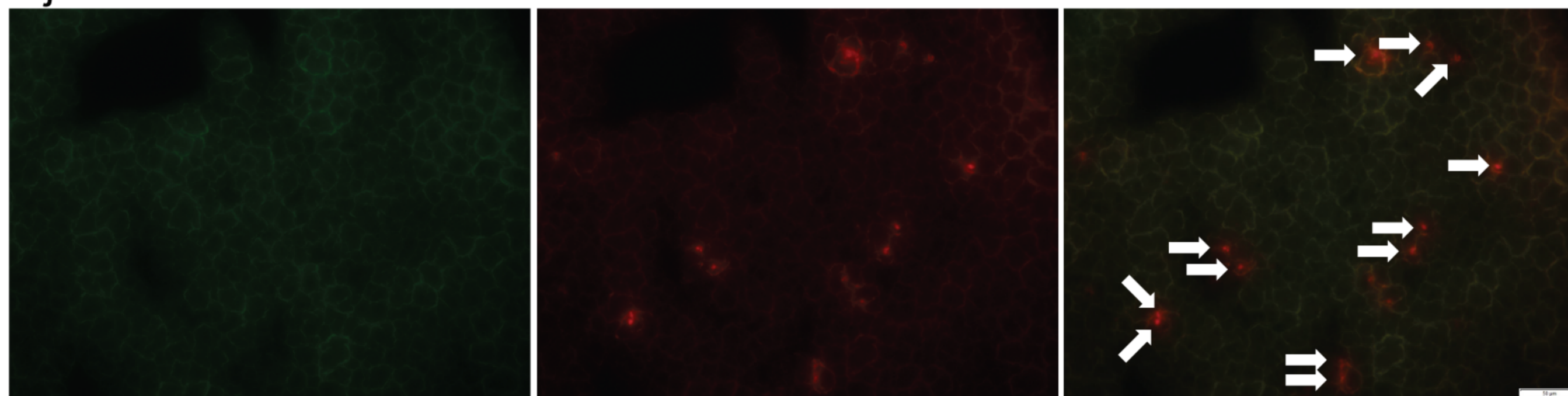

Non-injected Liver

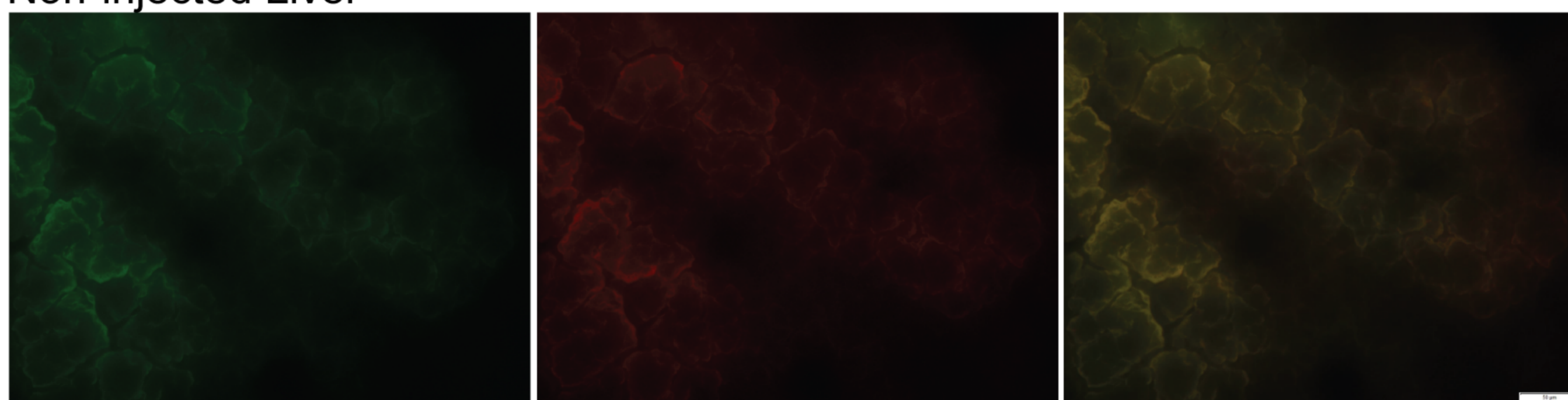

B Injected Uterus

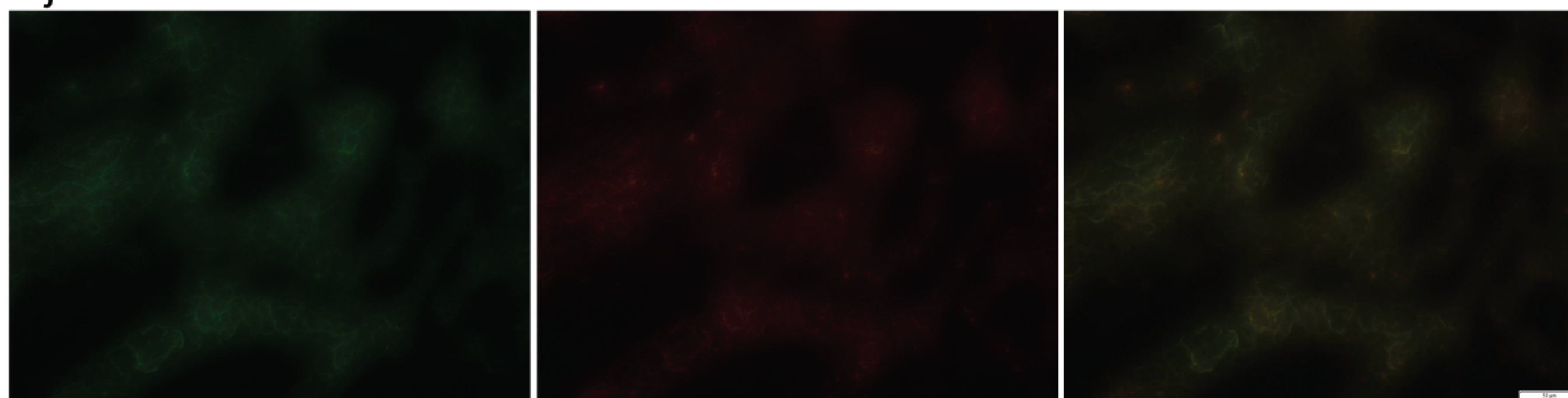

Non-injected Uterus

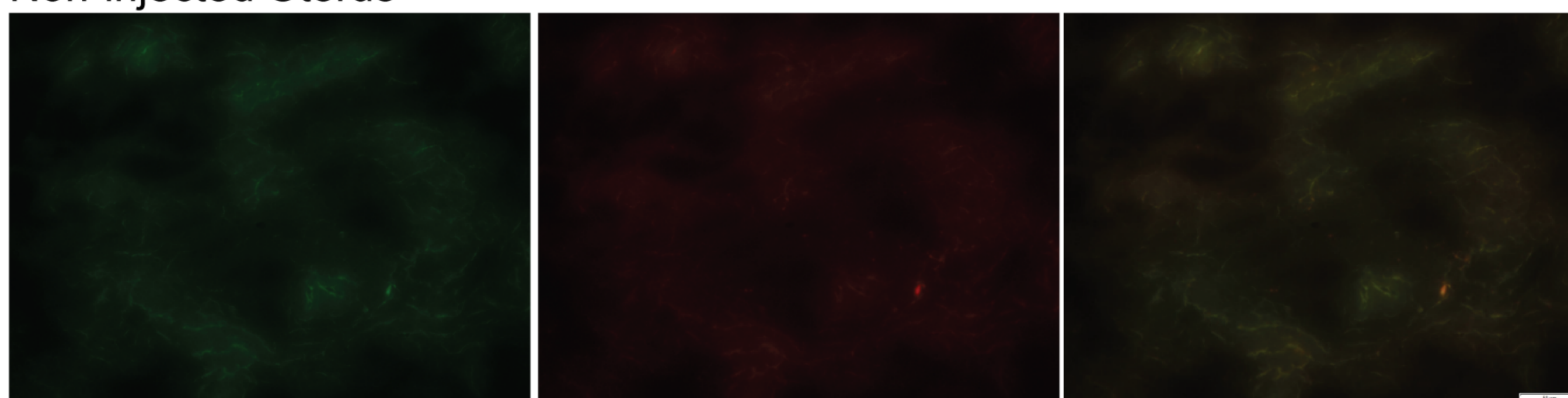

Supplement: S2 Fig — A) Photomicrographs of liver (A) and uterus (B) of non-pregnant Rag2-/- γc-/- mice injected (top) and non-injected (bottom) with 15 million CellTrakerTM Red CMTPX labeled idNK cells. Organs harvested 3 days post tail vein injection. Images obtained with a FITC filter (left), a Texas Red filter (center) and both channels superimposed (right). White arrows point to labeled cells. Bars represent 50μm. (PDF) [file pone.0164353.s002.pdf]
