# Supplementary material for: Induced Human Decidual NK-Like Cells Improve Utero-Placental Perfusion in Mice
Source: PLoS One. 2016 Oct 13;11(10):e0164353. doi: 10.1371/journal.pone.0164353 (PMC5063315; doi:10.1371/journal.pone.0164353)
Supplement: S1 Table — Supernatants from idNK and control pNK IL-15 collected at 7 days were measured for a panel of 46 cytokines, angiogenic factors and growth factors from two overlapping luminex kits. P-values shown are derived from two-tailed t-tests comparing the mean concentration of each analyte in supernatants of idNK cells vs. pNK cells from three donors. ** p < 0.01, *p < 0.05. pNK cells 24hs baseline expression correspond to fresh pNK cell from three independent donors cultured 24hs in the presence of IL15. (PDF) [file pone.0164353.s006.pdf]

MILLIPLEX® MAP Human Cytokine/Chemokine 30-Plex Panel				
Cytokine	idNK Mean Cytokine Expression (pg/ml)	pNK IL-15 Mean Cytokine Expression (pg/ml)	p-Value	pNK IL-15 Baseline Expression (pg/ml)
EGF	6.33±1.87	22.05±27.35	0.42	0.95±0.26
Eotaxin	6.08±3.04	0±4.74	*0.03	0.41±0.31
G-CSF	16.25±4.27	1.03±2.87	**0.005	6.93±2.90
GM-CSF	293.98±69.32	524.11±148.33	0.20	7.18±7.05
IFN2α	20.92±11.36	7.85±5.50	0.06	3.47±1.02
IFNγ	325.08±131.51	248.09±106.59	0.08	17.38±18.68
IL-10	1.30±1.13	0.84±0.89	0.34	0.94±0.42
IL-12p40	10.07±5.53	0±4.65	**0.008	2.80±1.69
IL-12p70	2.66±0.77	0±1.39	*0.03	0.24±0.31
IL-13	24.24±15.19	3.29±1.11	0.12	0.88±0.38
IL-17A	0.54±0.52	0.08±0.34	0.052	0.0001±0.03
IL-1RA	30.71±13.34	20.54±27.37	0.34	5.12±4.26
IL-1α	0.62±0.23	0.77±1.0	0.78	Not detected
IL-1β	1.26±0.65	0.49±0.54	*0.049	0.34±0.08
IL-2	1.77±1.04	0.18±0.43	*0.048	0.14±0.09
IL-3	Not detected	Not detected	N/A	Not detected
IL-4	5.26±3.34	2.21±2.02	0.08	0.80±0.79
IL-5	0.53±0.12	0.23±0.21	*0.04	0.08±0.05
IL-6	2.61±2.04	10.16±12.39	0.33	1.16±1.93
IL-7	Not detected	Not detected	N/A	0.44±0.21
IL-8	47.69±25.04	3.47±0.09	0.09	44.41±72.18
IP-10	48±31.21	297.37±424.36	0.40	5.05±3.29
MCP-1	20.47±3.82	6.43±8.75	0.19	90.60±152.84
MIP-1α	785.28±660.89	339.73±394.76	0.14	122.47±64.95
MIP-1β	4213.30±2505.47	1239.17±1365.6	0.052	130.78±81.74
RANTES	2823.20±2737.07	750.06±368.38	0.32	138.14±87.49
TNFα	111.96±19.57	180.91±33.40	0.15	5.20±2.24
TNFβ	6.08±2.23	9.62±1.44	0.12	1.58±0.79
VEGF	55.59±24.22	9.59±17.15	*0.04	Not detected
IL-1	Not detected	Not detected	N/A	Not detected
**MILLIPLEX® MAP Human Angiogenesis/Growth Factor 17-Plex Panel**				
Cytokine	idNK Mean Cytokine Expression (pg/ml)	pNK IL-15 Mean Cytokine Expression (pg/ml)	p-Value	pNK IL-15 Baseline Expression (pg/ml)
EGF	0.55±0.67	14.68±22.03	0.37	0±0.32
Angiopoetin-2	Not detected	Not detected	N/A	Not detected
G-CSF	Not detected	Not detected	N/A	Not detected

BMP-9	Not detected	Not detected	N/A	Not detected
Endoglin	1.69±0.25	5.58±1.3	*0.02	0.27±0.12
Endothelin-1	Not detected	Not detected	N/A	Not detected
Leptin	Not detected	Not detected	N/A	3.99±6.91
FGF-1	0±0.08	0.08±0.31	0.44	0±0.08
Follistatin	Not detected	Not detected	N/A	Not detected
HGF	Not detected	Not detected	N/A	Not detected
HB-EGF	0.67±0.41	9.85±0.47	**0.002	0.04±0.02
PLGF	0.58±0.44	0.23±0.14	0.198	Not detected
VEGF-A	947.93±142.1	55.46±48.75	**0.003	0.58±0.81
VEGFC	Not detected	Not detected	N/A	1.15±1.00
VEGFD	Not detected	Not detected	N/A	Not detected
FGF-2	Not detected	Not detected	N/A	Not detected
