# Supplementary material for: Induced Human Decidual NK-Like Cells Improve Utero-Placental Perfusion in Mice
Source: PLoS One. 2016 Oct 13;11(10):e0164353. doi: 10.1371/journal.pone.0164353 (PMC5063315; doi:10.1371/journal.pone.0164353)
Supplement: S2 Table — idNK conversion efficiency was evaluated by the percentage of CD9+KIR+cells in CD3-CD56BrightCD16- NK cell and in CD3-CD56DimCD16+ cell populations in the culture. Mice were injected with cells from the donor indicated in the table. (PDF) [file pone.0164353.s007.pdf]

idNK cells

Mouse	NK Donor	% of $CD9^+$ KIR^+ cells of $CD56^{Bright}$ idNK cells	% of $CD9^+$ KIR^+ cells of $CD56^{Dim}$ idNK cells
S1	A	32	54.9
S2	B	55.7	40.7
S3	B	55.7	40.7
S4	C	55	60
S5	D	55	60
S6	E	51.8	58.9

Control pNK cells maintained in culture in IL-15

Mouse	NK Donor	% of $CD9^+$ KIR^+ cells of $CD56^{Bright}$ NK cells	% of $CD9^+$ KIR^+ cells of $CD56^{Dim}$ NK cells
C1	E	8.1	17.8
C2	E	8.1	17.8
C3	F	7.5	8.1
C4	F	7.5	8.1
C5	F	7.5	8.1
C6	E	8.1	17.6

S2 Table. pNK to idNK conversion efficiency of cell preparations injected in pregnant mice.
